# *GRIN2B*-related neurodevelopmental disorders: genotype-phenotype correlations and therapeutic implications

**DOI:** 10.1186/s13023-025-04055-x

**Published:** 2025-10-27

**Authors:** Changning Xie, Miriam Kessi, Fangyun Liu, Fang He, Jing Peng

**Affiliations:** 1 Department of Pediatrics, Xiangya Hospital, Central South University, Xiangya Road 87, Changsha, Hunan 410008 China; 2 Hunan Intellectual and Development Disabilities Research Center, Changsha, Hunan 410008 China

**Keywords:** *GRIN2B*, Neurodevelopmental disorders, Phenotype-genotype associations, Gain-of-function, Loss-of-function

## Abstract

**Background:**

Pathogenic variants in *GRIN2B* are predominantly associated with neurodevelopmental disorders (NDDs). However, our understanding of the genotype-phenotype correlations and the optimal treatment strategies is limited.

**Methods:**

We collected clinical data of seven new patients from the Xiangya Hospital and conducted an extensive literature review. Subsequently, we carried out comparisons of clinical features between patients with gain-of-function (GOF) variants and patients with loss-of-function (LOF) variants, as well as patients with LOF missense variants and LOF truncating variants.

**Results:**

We identified seven patients, five of whom had novel variants (p.Phe554Ser, p.Val821Phe, p.Ile641Thr, p.Asn649Ser, and p.Gly1182Arg), and two of whom had known variants (p.Gly820Val and p.Met818Leu). Of the seven cases, 4 (57.14%) presented with epilepsy. Two of these individuals (carrying the p.Ile641Thr and p.Met818Leu variants, respectively) achieved seizure control after receiving memantine. The clinical manifestations included severe developmental delay /intellectual disability (DD/ID) and hypotonia in 100% of cases, as well as microcephaly in 28.57% of cases. Brain imaging results were available for six cases, five of which (83.33%) had abnormal results. Combining our patients (*n* = 7) and those reported in the literature (*n* = 98), a total of 105 patients were analyzed. These patients carried 84 pathogenic variants, ten of which were recurrent. The two most frequently occurring variants were p.Gly820Ala and p.Gly689Ser. The predominant phenotypes observed were DD/ID (100%), hypotonia (87.27%), epilepsy (53.08%), and language impairment (45.71%). We further analyzed the clinical genotypes and phenotypes of 55 patients with LOF variants and 16 patients with GOF variants. Variants located in the transmembrane domain predominantly resulted in a GOF effect. GOF variants were more likely to cause epilepsy and microcephaly than LOF variants. Moreover, in comparison to LOF truncated variants, missense variants were associated with more severe clinical phenotypes, including severe DD/ID, language delay, and movement disorders.

**Conclusions:**

This study reports five novel *GRIN2B* variants and seven additional patients. Notably, it is the first report to distinguish between GOF and LOF *GRIN2B* variants, which have distinct clinical phenotypes. Furthermore, memantine has been shown to be effective in controlling seizures and improving cognition.

**Supplementary Information:**

The online version contains supplementary material available at 10.1186/s13023-025-04055-x.

## Highlights

The predominant phenotypes associated with *GRIN2B* variants include developmental delay, intellectual disability, hypotonia, epilepsy, and language impairment.

Variants located in the transmembrane domain predominantly lead to a gain-of-function (GOF) effect.

GOF variants are more likely than LOF variants to cause epilepsy and microcephaly.

In comparison to LOF-truncated variants, missense variants result in more severe clinical phenotypes.

Approximately 25.93% of patients can achieve seizure control.

Memantine can control seizures and improve cognitive function.

**Supplementary Information:**

The online version contains supplementary material available at 10.1186/s13023-025-04055-x.

## Introduction

N-methyl-D-aspartate (NMDA) receptors are di- or triheterotetrameric ligand-gated ion channels [1]. They are composed of two obligate glycine-binding GluN1 subunits and two glutamate-binding GluN2 or GluN3 subunits [1]. These subunits are encoded by the *GRIN1*, *GRIN2A-D*, and *GRIN3A-B* genes [1]. Each NMDAR subtype displays distinct temporal and spatial expression patterns in the brain [1]. At the sub - cellular level, they are specifically localized to different cell types, which gives rise to a diverse spectrum of protein functions [1]. All NMDAR subunits consist of four domains: the amino-terminal domain (ATD), the agonist-binding domain (ABD), the transmembrane domain (TMD), and the carboxyl-terminal domain (CTD) [1]. The ATD is involved in subunit assembly and allosteric modulation [2]. The TMD is made up of four segments (M1, M2, M3, and M4), with M2 reentrant forming the pore loop [2]. The CTD is involved in the process of receptor trafficking [2]. Glycine and glutamate bind to the ABD of the GluN1 and GluN2 (A–D) subunits, respectively. These binding events regulate the opening of the ion channels [2].

NMDARs play a critical role in mediating excitatory neurotransmission and are essential for regulating neuronal development and synaptic plasticity [3]. These receptors are essential for learning, memory, and cognitive function [3]. Among the NMDAR subunits, GRIN1, GRIN2A, GRIN2B, and GRIN2D are primarily implicated in human neurological and psychiatric diseases [4–7]. Specifically, genetic defects in *GRIN2B* are primarily correlated with neurodevelopmental disorders, including developmental delay (DD), intellectual disability (ID), and autism spectrum disorder (ASD), as well as schizophrenia-like phenotypes [1]. However, there is a significant gap in our understanding of genotype-phenotype correlations and the most effective strategies.

Therefore, to elucidate the genotype-phenotype correlations of *GRIN2B*-related neurological disorders and the most effective treatment options, we present the phenotypes, therapies, and neurophysiological profiles of seven new patients harboring five novel and two previously reported *GRIN2B* pathogenic variants. Additionally, we conducted a systematic literature review of all reported *GRIN2B* pathogenic and likely pathogenic variants. We then summarized their associated clinical phenotypes, electroencephalography (EEG) findings, brain magnetic resonance imaging (MRI) results, as well as the effects of anti-seizure medications (ASMs) and other therapeutic approaches. The benefits of memantine and adrenocorticotropic hormone (ACTH) therapies have been emphasized. Notably, this is the second study to report on the efficacy of memantine in *GRIN2B*-related neurodevelopmental disorders. This study will improve the clinical understanding of this gene and provide guidance for diagnosis and treatment.

## Methods

The study was approved by the Ethics Committee of Central South University in China (protocol/human study approval number 201603205) and was performed in accordance with the ethical standards set forth in the Declaration of Helsinki. Written informed consent for publication was obtained from the patients’ parents or guardians.

### Participants

Between June 2015 and October 2024, patients with *GRIN2B* variants were recruited from Xiangya Hospital, Central South University. Epileptic seizures and syndromes were defined according to the International League Against Epilepsy (ILAE) classification. Other neurodevelopmental disorders, including DD, ID, and ASD, were diagnosed using the Diagnostic and Statistical Manual of Mental Disorders, Fifth Edition (DSM-5). ID assessments were performed based on the DSM-5 diagnostic criteria as described in our previous studies [8, 9]. We used observations, clinical interviews, and standardized, age-related rating scales to assess adaptive functioning. However, the diagnosis was often initially formulated based on clinical judgment rather than formal, standardized assessments, especially for young patients [10]. The standardized age-related rating scales used were: the Gesell Developmental Schedules for patients aged 2–4 years; the Wechsler Preschool and Primary Scale of Intelligence Fourth Edition (WPPSI-IV) for patients aged 4–6 years; and the Wechsler Intelligence Scale for Children Fourth Edition (WISC-IV) for patients aged 6 years old and above. For patients younger than 2 years old, clinical judgment was used to grade the severity of DD. Senior pediatric neurologists, geneticists, EEG technicians, and radiologists attended to all patients. The clinical information of five patients, including seizure types, EEG abnormalities, developmental milestones, MRI findings, and response to ASMs, was collected retrospectively from the hospital database. The clinical information of the remaining two patients who received memantine treatment was collected prospectively. Physicians and parents assessed the cognitive improvement of patients before and after therapies. We compared neurological signs, including behavior, attention, eye contact, and social interaction, before and during memantine treatment as described before [11]. All patients identified with *GRIN2B* variants were followed up at an outpatient setting or via telephone every three months.

### Genetic analyses

*GRIN2B* (NM_000834) variants were identified by whole exome sequencing of peripheral blood deoxyribonucleic acid (DNA) from patients and family members, using the standard methods previously described [12, 13]. After assessing the frequency of the identified variants in the general population using genomAD, variants were classified as pathogenic or likely pathogenic. Additionally, prior to classification, the effect of the variant on protein function was assessed using several predictors: PolyPhen2, SIFT, and Mutation Taster. Candidate variants were validated and confirmed using Sanger sequencing. The variants were interpreted according to the standard guidelines of the American College of Medical Genetics and Genomics (ACMG) [14].

As described previously, nonsense, deletion, frameshift, splice, and translocation variants were classified as loss-of-function (LOF) variants [15]. Missense variants were classified as LOF or gain-of-function (GOF) based on functional experiments, as described in a previous study [15].

### Literature review

A comprehensive literature review was conducted in PubMed covering all years up to August 29, 2024. The search terms were “GRIN2B” and “epilepsy” or “neurodevelopmental disorder” or “developmental delay” or “intellectual disability” or “autism spectrum disorder”. Only articles written in English were collected and filtered. The reference lists of all the identified articles were also checked to ensure that no important papers were missed. The severity of DD/ID was categorized based on the primary authors. All reported variants were classified as either GOF or LOF based on the patch clamp experiments results from the literature.

### Statistics

A comparison of clinical features was carried out between two groups of patients: one with GOF variants and the other with LOF variants. We also compared the clinical features of patients with LOF missense variants to those with LOF protein-truncating variants (nonsense, frameshift, and splicing). We used the chi-square test (χ²) or Fisher’s exact test to compare differences between two sample sets. A p-value of less than 0.05 was considered statistically significant. The GraphPad Prism 7.0 and SPSS 22.0 software programs were used to process and analyze the data.

## Results

We identified 98 individuals with pathogenic or likely pathogenic *GRIN2B* variants from 23 publications [11, 16–37]. Additionally, we reported the clinical data for seven novel cases harboring likely pathogenic or pathogenic *GRIN2B* variants.

### Patients with GRIN2B variants from our center

We recruited seven new cases from our center, four of which were female. These seven cases carried five novel variants: p.Phe554Ser, p.Ile641Thr, p.Asn649Ser, p.Val821Phe, and p.Gly1182Arg as well as two previously published variants: p.Met818Leu and p.Gly820Val [11, 22]. All seven variants were *de novo* and were classified as likely pathogenic or pathogenic according to the ACMG guidance. Four variants were located in the M4 transmembrane helix, two in the M3 transmembrane helix, and one in the M1 transmembrane helix. Of the 7 cases, 4 (57.14%) patients presented with epilepsy. Of these patients, only one patient who carried the p.Ile641Thr variant achieved seizure freedom after receiving memantine. All seven cases exhibited severe DD/ID and hypotonia. Microcephaly was observed in two cases (28.57%). Brain MRI results were available for six cases, five of which (83.33%) had abnormal results. Table 1 summarizes this data.

**Table 1:** Genetic and clinical characteristics of cases carrying *GRIN2B* variants from our hospital

Case	1	2	3	4	5	6	7
Current Age/Sex	7 y/F	4 y 8 mo/M	3 y/F	11 mo/M	9 mo/F	4 y/M	3 y 3 mo/F
cDNA change	c.1661T > C	c.1922T > C	c.1946 A > G	c.2452 A > T	c.2459G > T	c.2461G > T	c.3544G > A
Protein change (NM-000834)	p.Phe554Ser	p.Ile641Thr	p.Asn649Ser	p.Met818Leu	p.Gly820Val	p.Val821Phe	p.Gly1182Arg
Domain	M1	M3	M3	M4	M4	M4	M4
Inheritance	*De novo*	*De novo*	*De novo*	*De novo*	*De novo*	*De novo*	*De novo*
Novel/ Published	Novel	Novel	Novel	Published [22]	Published [11]	Novel	Novel
ACMG scores	PS2 + + PM2 + PP2 + PP3	PS2 + PM2 + PP2 + PP3	PS2 + + PM2 + PP2 + PP3	PS2 + PS3 + PP2 + PP3	PS2 + PS3 + PP2 + PP3	PS2 + PM2 + PP2 + PP3	PS2 + PM2 + PP2 + PP3
Final ACMG classification	LP	LP	LP	P	P	LP	LP
Seizures	No Seizures	Infantile spasms	No seizures	Epileptic spasms	Infantile spasms	No seizures	No seizures
EEG features	Spike-slow waves occured in the bilateral frontal brain. These waves increase during sleep.	Slow background rhythm with spike and sharp waves in the right central, frontal, and temporal regions.	Slow background, spike waves, and sharp waves in the posterior head, especially on the left.	Spike waves and sharp waves in the temporal and occipital regions were prominently seen on the right.	Background: a large amount of beta activity was observed in all regions with intermittent release in central or generalized theta rhythms.	Spike waves and slow waves in the left temporal and occipital regions were present.	NA
ASMs	No drug	OXC, VPA, ACTH (partial improvement), memantine (seizure free)	Clonazepam	LEV, PB, TPM, L-serine, perampanel (no improvement), memantine (improvement)	TPM (no improvement), ACTH (partial improvement)	No drug	No drug
DD/ID	Severe GDD	Severe GDD	Severe GDD	Severe GDD	Severe GDD	Severe GDD	Severe GDD
Current development	She cannot sit or speak	He cannot sit or speak	She cannot walk independently and she can only few words	He cannot sit and he has severe language developmental delay	She cannot sit and she has severe language developmental delay	He cannot sit or speak	She has unsteady walk
Other neurological features	Hypotonia	Fine motor disorder, hypotonia, and microcephaly	Hypotonia	Hypotonia	Ptosis in the left eye, hypotonia, and microcephaly	Hypotonia	Hypotonia
Magnetic resonance imaging	There is widening of the ventricles in the bilateral frontotemporal regions.	There is a widened subarachnoid space in the bilateral frontotemporal regions.	There is a widened ventricles	There is widening of the bilateral frontotemporal sulci and the temporal horn of the right ventricle.	Normal	Bilateral temporal and frontal lobe hypoplasia.	NA
Additional findings	Vaginal dysplasia and feeding difficulties	Normal	Normal	Visual impairment	NA	NA	NA

Abbreviations: NA: not available; mo: months; EEG: electroencephalograph; GDD: global developmental delay; ID: intellectual disability, ASMs: anti-seizure medications; P: pathogenic; LP: likely pathogenic; OXC: oxcarbazepine; VPA: sodium valproate; ACTH: Adrenocorticotropic hormone; LEV: levetiracetam; PB: phenobarbital; TPM: topiramate; VUS: variant of unknown significance. Y: year; M: male; F: female

### Clinical characteristics of individuals with reported and novel GRIN2B likely pathogenic or pathogenic variants

After combining our novel variants and those reported in the literature, we found 105 cases with *GRIN2B* variants. Information about gender was available for 76 cases, 40 (52.63%) of which were males. The mean age at inclusion and publication was 5.7 (range, 0–30) years. Of the 81 patients with available epilepsy-related information, 53.08% (43/81) presented with seizures. The mean age at seizure onset was 22.69 (range 0–108) months. The types of seizures included generalized seizures (13/36, 36.11%), focal seizures (15/36, 41.67%), infantile spasms (9/36, 25%), and epileptic spasms (9/36, 25%). Information about seizure outcomes was available for 27 patients. Of those patients, 25.93% (7/27) achieved seizure control. Abnormal EEG results were found in 87.93% (51/58) of cases. Of the 105 cases, all (100.00%) presented with DD/ID. Of those, 71 (67.62%) cases had known severity. Among the individuals with known DD/ID severity, mild manifestations were observed in 15.49% (11/71) of the cases, moderate manifestations in 19.72% (14/71), severe manifestations in 63.38% (45/71). Of the 81 patients for whom information about ASD was available, 25 (30.86%) patients had it. Other clinical manifestations included movement and motor disorders (43/105, 40.95%), spasticity (6/41, 14.63%), hypotonia (48/55, 87.27%), microcephaly (10/55, 18.18%), and language impairment (48/105, 45.71%). Among individuals with language impairment, 64.58% (31/48) could not speak. Brain MRI results were available for 62 cases, 25 of which had abnormal results, including widened ventricles (*n* = 6), polymicrogyria (*n* = 5), delayed myelination (*n* = 4), and hypoplastic corpus callosum (*n* = 4). Other anomalies included an abnormal cerebellum (*n* = 2), cortical atrophy or dysplasia (*n* = 4), basal ganglia dysplasia (*n* = 3), an abnormal signal in the left amygdala (*n* = 1), a widened subarachnoid space in the bilateral frontotemporal regions (*n* = 1), and increased extra-axial spaces (*n* = 1). Supplementary Table 1 summarizes these results.

A total of 84 likely pathogenic or pathogenic variants were identified in the 105 cases. Of the 84 variants identified, ten were recurrent: p.Gly820Ala (*n* = 6), p.Gly689Ser (*n* = 6), p.Arg847* (*n* = 4), p.Ile751Thr (*n* = 3), and p.Met818Leu (*n* = 2), p.Ala734Val (*n* = 2), p.Arg540His (*n* = 2), p.Arg696His (*n* = 2), p.Gly820Val (*n* = 2), and p.Lys1091Thr (*n* = 2). There were 69 missense variants and 16 truncated variants (Supplementary Table 1). The p.Met818Leu variant was observed in two males presenting with early onset epileptic spasms, severe DD, hypotonia, and visual impairment. One of the patients was seen at our center. He was treated with levetiracetam (LEV), phenobarbital (PB), topiramate (TPM), L-serine, and perampanel; however, he continued to have refractory epilepsy. The second patient was found in the literature and was treated with LEV, clobazam (CLB), TPM, and prednisolone, but did not improve. However, memantine reduced seizure frequency by 80%. The p.Gly820Ala variant was found in six patients presenting with heterogeneous phenotypes: DD/ID (*n* = 6), ASD (*n* = 2), movement disorders (*n* = 2), and refractory infantile spasms (*n* = 1). The patient with infantile spasms was treated with valproic acid (VPA), which did not improve the condition. Although carbamazepine (CBZ) led to a temporary improvement, the patient remained with uncontrolled seizures. The p.Arg847* variant was identified in four individuals with various manifestations: DD/ID (*n* = 4), ASD (*n* = 1), and hypotonia (*n* = 2). The p.Gly689Ser variant was found in six individuals presenting with DD/ID (*n* = 6), hypotonia (*n* = 4), movement disorders (*n* = 4), and seizures (*n* = 3). One patient with a seizure disorder received treatment with vigabatrin (VGB), which increased seizure frequency. Treatment with TPM led to a partial improvement, while the patient still experiences ongoing seizures. The p.Ile751Thr variant was found in three cases who presented with DD/ID (*n* = 3), ASD (*n* = 1), and hypotonia (*n* = 3).

### Clinical features of patients with LOF variants and GOF variants, and clinical indicators for LOF and GOF variants

Electrophysiological channel characterization was conducted for 41 out of 85 (48.24%) of all reported *GRIN2B* variants. Previous studies have described 42 different LOF electrophysiological variants, including 18 in the ABD domain, seven in the TMD, and three in the CTD [11, 21, 34, 36, 38–41]. Previous studies [11, 39], found that thirteen different variants caused a GOF effect in cellular expression systems, including five variants in the ABD domain and eight in the TMD domain. Furthermore, variants in the TMD domain were more likely to be GOF (*P* < 0.05). As previously proposed, we grouped patients according to the known functional consequences on NMDARs for phenotypic comparison (Table 2, Supplementary Table 2).

To date, 42 different *GRIN2B* LOF variants have been identified in 55 cases, and 14 different GOF variants have been identified in 16 cases. Of the 55 patients with LOF variants, 48 had information about epilepsy. Of those, 41.67% (20/48) experienced seizures. The mean age at seizure onset was 12 months (range: 0–108). Types of seizures included generalized (5/14, 35.71%), focal (3/14, 21.43%) seizures, both focal and generalized (2/14, 14.29%), myoclonic seizures (4/14, 28.57%), and infantile spasms (1/14, 7.14%). Information about seizure outcomes was available for 17 patients. Of those patients, 29.41% (5) achieved seizure control. Abnormal EEG results were found in 82.35% (28/34) of cases. All 55 cases (100.00%) presented with DD/ID. Of the 38 cases with known DD/ID severity, mild manifestations were observed in seven (18.42%) patients, moderate in eight (21.05%) patients, and severe in 23 patients (60.53%). ASD was observed in 29.00% (16/55) of patients. Additional clinical manifestations included movement and motor disorders (45.45%, 25/55), language impairment (49.09%, 27/55), and microcephaly (14.29%, 4/28). Of the patients with language impairment, 59.26% (16/27) could not speak. Brain MRI results were available for 35 cases, 13 of which had abnormal results, including widened ventricles, delayed myelination, cortical atrophy, and hypoplastic corpus callosum. In addition, LOF missense variants were found to be associated with more severe clinical phenotypes than LOF truncated variants. These severe clinical manifestations included severe DD/ID, speech impairment, and movement disorders (Table 3, Supplementary Table 5).

**Fig. 1:**
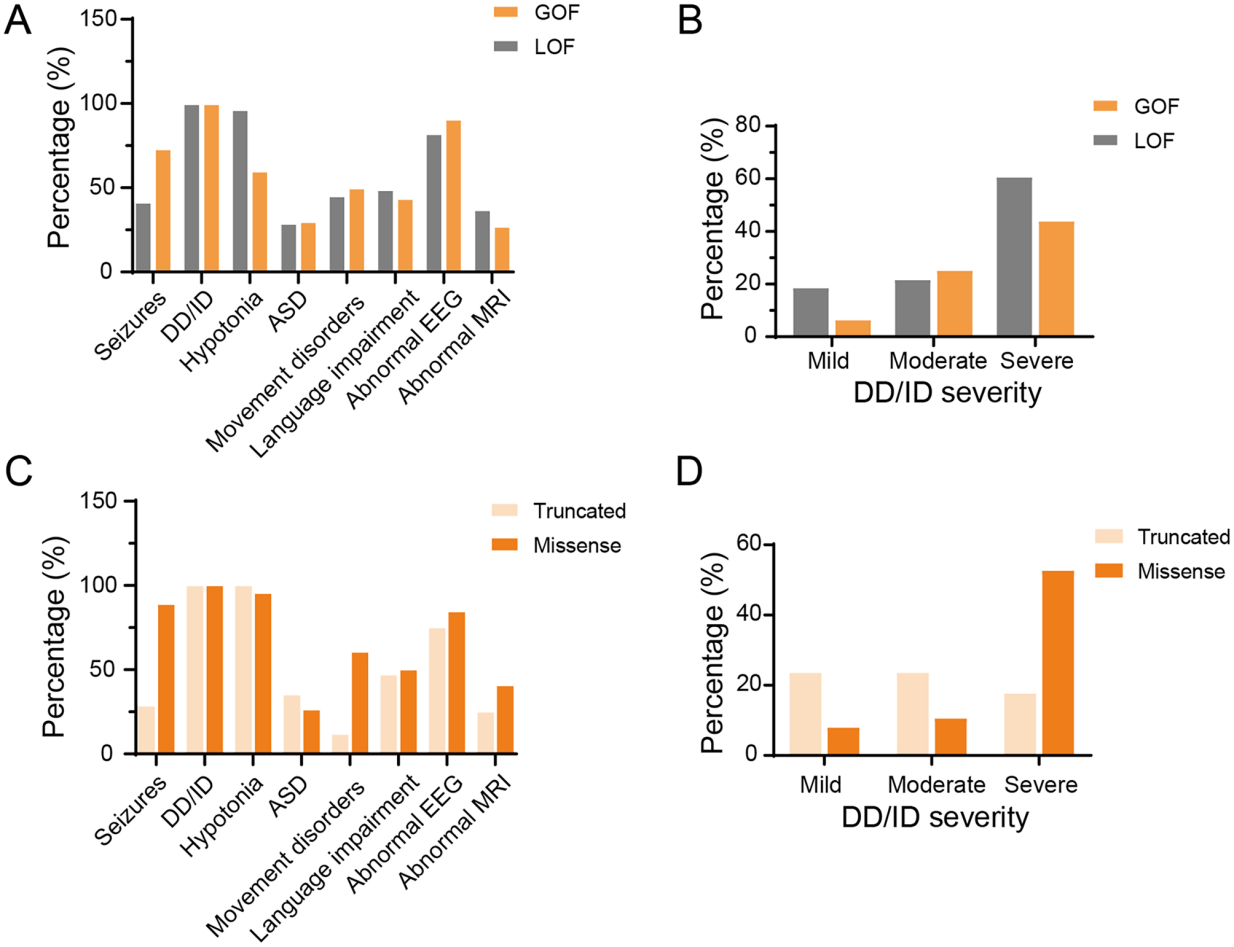
Distinctive phenotypic features of GRIN2B-related neurodevelopmental disorders in individuals with different type of variants. (**A**, **B**) Representation of the main phenotypic features in patients carrying GOF versus LOF variants. Values are presented as the percentage of patients exhibiting each trait, calculated relative to the number of patients with available data. (**C**, **D**) Representation of the main phenotypic features in patients carrying LOF missense versus truncated variants. Abbreviations: DD: developmental delay, ID: intellectual disability, EEG: electroencephalograph, ASD: autism spectrum disorder, MRI: magnetic resonance imaging

Of the 16 patients with GOF variants, 15 had information about epilepsy, 11 of whom (73.33%) presented with seizures. The mean age at seizure onset was 3.5 (range, 0-117) months. The types of seizures included generalized seizures (2/10, 20%) and focal seizures (1/10, 10%). Of the eleven patients with information about seizure outcomes, only one achieved seizure control. The EEG was abnormal in 90.91% (10/11) of cases. All 16 cases (100%) presented with DD/ID. Of the 12 cases with known DD/ID severity, one patient had a mild form, four patients had a moderate form, and seven patients had a severe form. Five out of 15 patients (33.33%) presented with ASD. Additional clinical manifestations included movement and motor disorders (8/16, 50%), language impairment (7/16, 43.75%), and microcephaly (5/16, 31.25%). Of the patients with language impairment, 85.71% (6/7) had no speech. Brain MRI results were available for 11 cases, 3 of which (27.27%) had abnormal results, including severe cortical and central atrophy, delayed myelination, and polymicrogyria of the optic nerves and optic chiasm. Figure 1 summarizes these results.

Compared to individuals with LOF variants, patients with GOF variants were more likely to experience seizures of various severities and microcephaly (*p* < 0.05). However, there was no significant difference in seizure onset, seizure frequency, or epilepsy outcome among the groups. Furthermore, no substantial disparities were observed between the two groups in other phenotypes, encompassing DD/ID, language impairment, movement disorder, ASD, and brain MRI and EEG abnormalities (Table 2).

**Table 2:** Comparison of clinical features between patients with GOF variants and those with LOF variants

Variable	LOF (*N* = 55)	GOF (*N* = 16)	*P* value
Variants	42	14	
Sex	Males (*n* = 20); females (*n* = 20)	Males (*n* = 8); females (*n* = 8)	-
Domain	ATD (8); S1 (5); L2 (5); M1 (2); M2 (1); L4 (1); M3 (1); L5 (2); S2 (10); M4 (4); CTD (3)	S1(2); M2(5); M3(1); L6(1); S2(2); M4(3)	ATD + ABD: *P* = 0.02*; TMD: *P* = 0.003*
Seizure	Yes (20); no (28)	Yes (11); no (4)	*P* = 0.04*
Median seizure onset age (months)	12 (0.03–108)	3.5 (0.1–117)	Survival curve: Log Rank: *P* = 0.98
Seizure types	Focal only (3/14)	Focal only (1/10)	-
	Generalized only (5/14)	Generalized only (2/10)	
	Both focal and generalized (2/14); myoclonic seizures(4/14), infantile spasms (1/14)	Both focal and generalized (2/10); infantile spasms (3/10)	
Seizure freedom	5/17	1/11	*P* = 0.36
Presence of DD/ID and severity	Yes (55/55); mild (7/38); moderate (8/38); severe (23/38)	Yes (16/16); mild (1/16); moderate (4/16); severe (7/16)	Mild ID: *P* = 0.41; moderate: *P* = 0.73 severe ID: *P* = 0.37
Language impairment	Yes (27/55), no speech (16/27)	Yes (7/16), no speech (6/7)	No speech: *P* = 0.38
Movement disorder	Yes (25/55)	Yes (8/16)	*P* = 0.78
ASD	Yes (16/55)	Yes (5/15)	*P* = 0.76
Hypotonia	Yes (28/29)	Yes (6/10)	*P* = 0.01*
Microcephaly	Yes (4/28)	Yes (5/10)	*P* = 0.04*
MRI	Abnormal (13/35); normal (22/35)	Abnormal (3/11); normal (8/11)	*P* = 0.72
EEG	Abnormal (28/34); normal (6/34)	Abnormal (10/11); normal (1/11)	*P* = 0.66

Abbreviations: ASD: autism spectrum disorder; EEG: electroencephalograph; DD: developmental delay; ID: intellectual disability; LOF: loss-of-function; GOF: gain-of-function; MRI: magnetic resonance imaging. * indicates *P* < 0.05

**Table 3:** Comparison of clinical features between the patients with LOF missense variants vs patients with LOF protein truncating variants

Variable	Patients with truncated variants (*N* = 17)	Patients with missense variants (*N* = 38)	*P* value
Number of variants	16	26	
Sex	Males (6); females (8)	Males (14); females (12)	-
Domain	ATD (8); M1 (1); L5 (1); S2 (4); CTD (2)	S1 (5); L2 (5); M1 (1); M2 (1); L4 (1); M3 (1); L5 (1); S2 (6); M4 (4); CTD (1)	ATD + ABD: *P* = 0.49; TMD: *P* = 0.13
Seizure	Yes (4); no (10)	Yes (16); no (18)	*P* = 0.34
Median seizure onset age (months)	22 months (6-108 months)	12 months (0–90 months)	Survival curve: Log Rank: *P* = 0.98
Seizure types	Focal only (3/3)	Focal only (2/11)	-
	Generalized (1/3)	Generalized (7/11)	
		Myoclonic seizures (4/11), infantile spasms (1/11)	
Seizure freedom	2/8	3/9	*P* > 0.99
Presence of DD/ID and severity	Yes (17/17); mild (4/17); moderate (4/17); severe (3/17)	Yes (38/38); mild (3/38); moderate (4/38); severe (20/38)	Mild ID: *P* = 0.19; moderate: *P* = 0.24 severe ID: *P* = 0.02*
Language impairment	Yes (8/17), no speech (2/9)	Yes (19/38), no speech (14/19)	No speech: *P* = 0.017*
Movement disorder	Yes (2/17)	Yes (23/38)	*P* = 0.001*
ASD	Yes (6/17)	Yes (10/38)	*P* = 0.53
Hypotonia	Yes (7/7)	Yes (21/22)	*P* > 0.99
Microcephaly	Yes (1/7)	Yes (3/22)	*P* > 0.99
MRI	Abnormal (2/8); normal (6/8)	Abnormal (11/27); normal (16/27)	*P* = 0.68
EEG	Abnormal (6/8); normal (2/8)	Abnormal (22/26); normal (4/26)	*P* = 0.61

Abbreviations: ASD: autism spectrum disorder; EEG: electroencephalograph; DD: developmental delay; ID: intellectual disability; LOF: loss-of-function; GOF: gain-of-function; MRI: magnetic resonance imaging. * indicates *P* < 0.05

### Treatment of GRIN2B-related epilepsy: conventional anti-seizure medications and memantine

Treatment information was available for 26 cases, 21 of which were from the previous study and five of which were from this study (Supplementary Tables 3, 4). Of the 26 patients, 10 patients (38.46%) were refractory to ASMs, five patients (19.23%) had a partial response, and 11 patients (42.31%) achieved seizure freedom. The seizure freedom proportions for effective drug combinations were as follows: ACTH/prednisolone (3/3); lamotrigine (1/1); PB (1/1); TPM (1/4); VPA (0/4); oxcarbazepine (0/2); CBZ (0/1); sulthiame (0/1); CLB (0/1); VGB (0/1); midazolam (0/1); and LEV (0/1). The efficacies of the therapies used for epilepsy, either alone or in combination, were as follows: ACTH/prednisolone (60%, 3/5); TPM (30%, 3/10); LTG (40%, 2/5); VPA (8.33%, 1/12); CBZ (25%, 1/4); VGB (14.29%, 1/7); PB (33.33%, 1/3); and L-serine (50%, 1/2). Furthermore, rufinamide and the ketogenic diet were reported to be effective in one case each. However, LEV, perampanel, midazolam, CLB, and OXC were ineffective (Fig. 3). Of the nine patients who presented with infantile spasms, three were administered ACTH. Two of these patients (66.67%) achieved partial seizure control, while one patient (33.33%) showed a poor response, likely due to the presence of status epilepticus. We administered ACTH to our two patients diagnosed with infantile spasms. However, they experienced only a transient reduction in seizures, with no cognitive or developmental improvement.

**Fig. 2:**
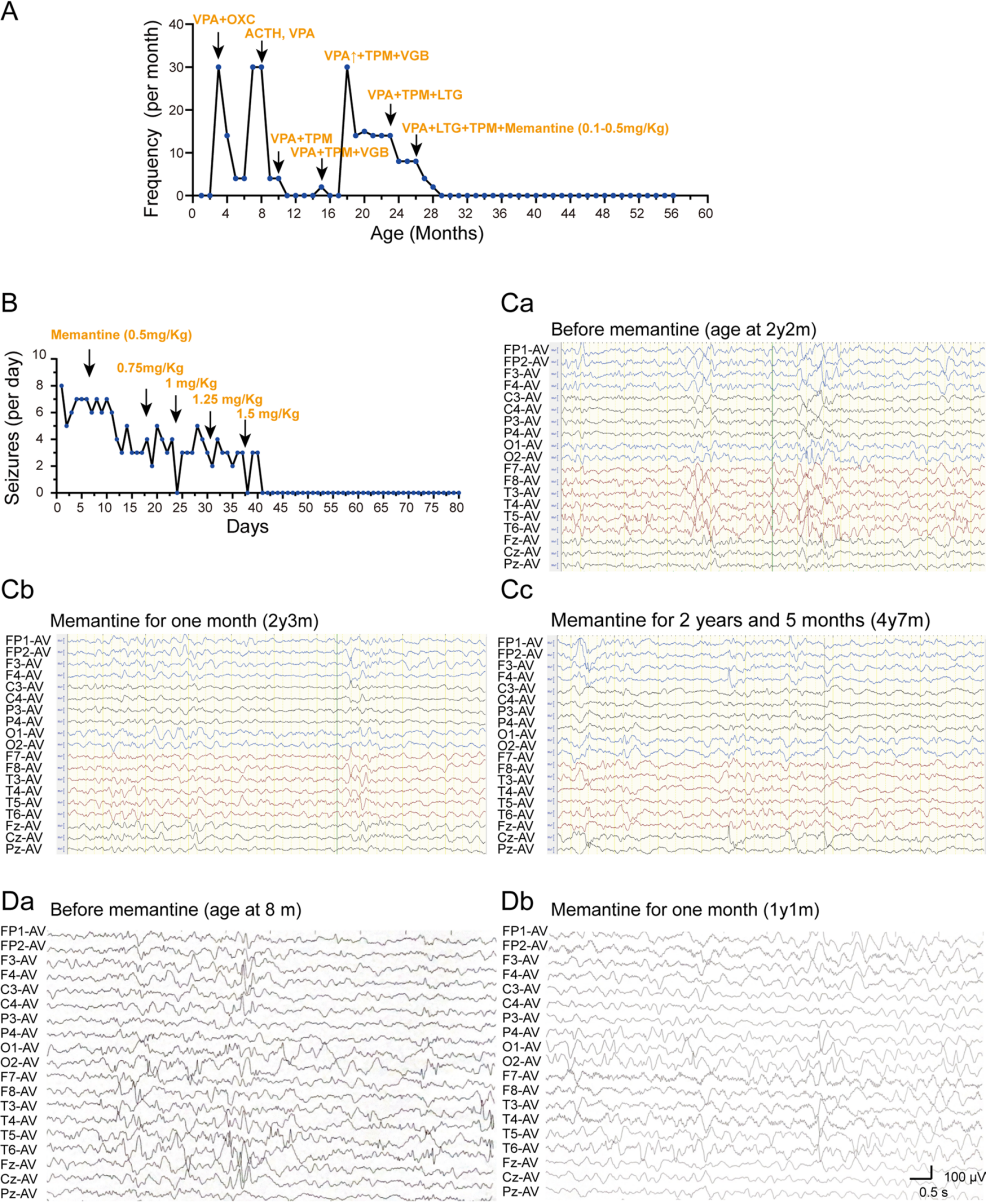
The efficacy of anti-seizure medications and memantine was assessed in two patients harboring distinct variants in the TMD region. (**A**, **B**) Seizure counts for the patients with the p.Ile641Thr and p.Met818Leu variants, respectively. (**Ca**-**Cc**) Electroencephalographic (EEG) recordings of patients carrying the p.Ile641Thr variant before (**Ca**) and after the application of memantine (**Cb**, **Cc**). All three EEG recordings show multifocal spike waves, spike-and-sharp waves, slow-spike waves, and sharp waves, prominently in the posterior regions. Post memantine EEGs (**Cb**, **Cc**) demonstrate a significant reduction in spike waves compared with **Ca**. Notably, epileptic spasms were observed in the pre-treatment EEG. (**Da**-**b**) EEG recordings of patients carrying the p.Met818Leu variant before (**Da**) and after (**Db**) the application of memantine. **Da**, EEG recordings show multifocal spike waves, sharp waves, slow-spike waves, and sharp waves predominantly in occipital and temporal regions. **Db**, EEG of post memantine reveals a slight reduction in spike waves when compared to **Da**

**Fig. 3:**
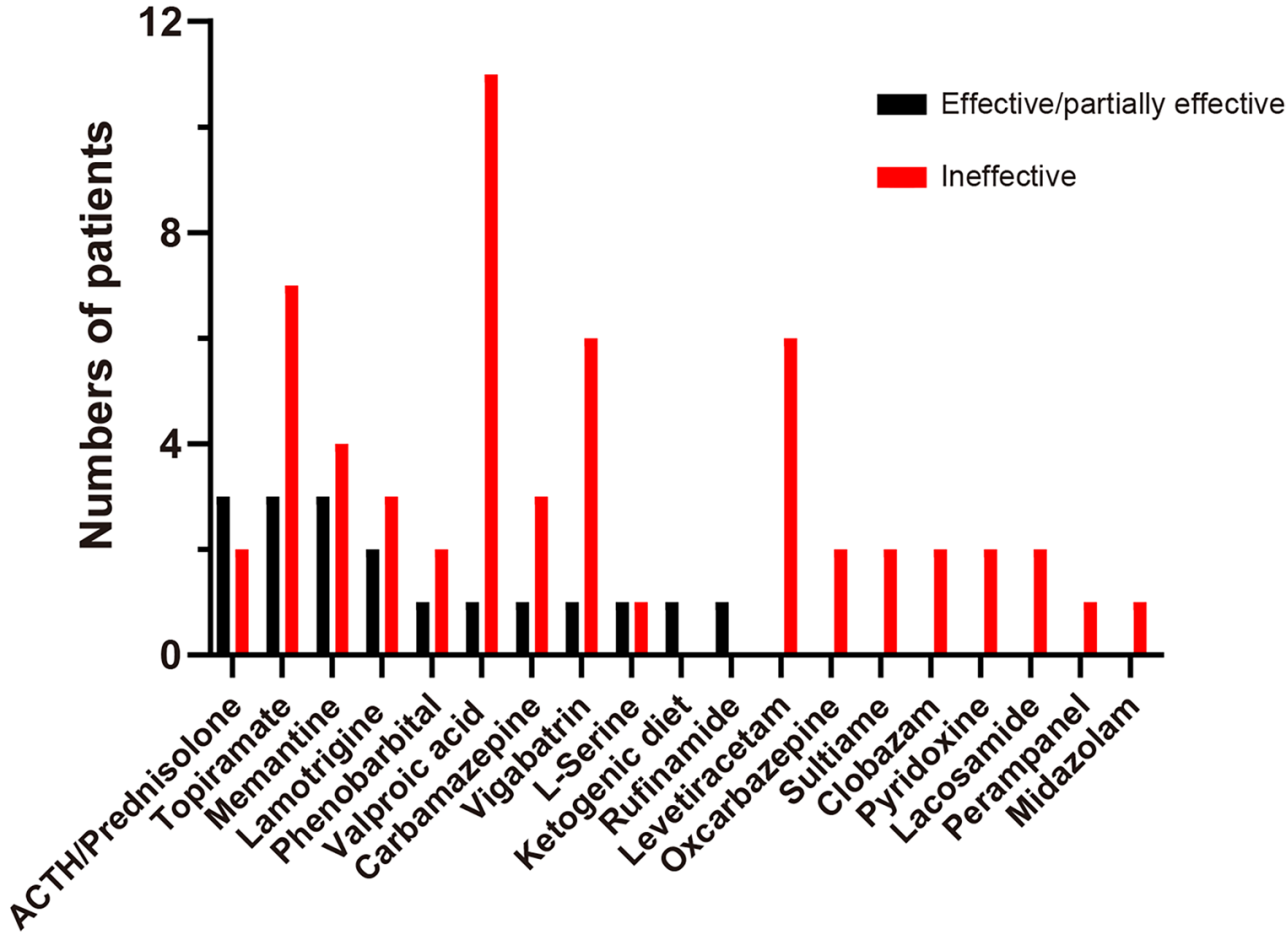
The bar graph summarizes the treatments used for epilepsy and their efficacy. Abbreviations: ACTH, adrenocorticotropic hormone

Besides, memantine effectively controlled seizures and improved cognition in patients with *GRIN2B*-related developmental disorders. Two patients in our cohort received memantine treatment, which resulted in seizure control and EEG normalization (Fig. 2). In addition, we observed cognitive improvement in some domains, including attention, eye contact, and social interaction in our two patients. In patient 2, a two-month memantine regimen elicited marked improvements in social reciprocity, as evidenced by enhanced social smiling and sustained attention. For patient 4, memantine administration resulted in partial responsiveness to auditory stimuli and slight gains in attention. None of our patients experienced adverse drug effects. Of the seven patients (including those from the literature) who were treated with memantine, six experienced cognitive improvement (85.71%), and three achieved seizure control (42.86%) (Fig. 3 summarizes this information).

## Discussion

An increasing use of next-generation sequencing technologies has led to a greater number of individuals being diagnosed with *GRIN2B* variants, broadening the phenotypic spectrum. In this study, we identified five novel *GRIN2B* variants, thereby expanding its genotypic spectrum. Five of the identified variants: p.Phe554Ser, p.Val821Phe, p.Ile641Thr, p.Asn649Ser, and p.Gly1182Arg variants were *de novo*. By incorporating all cases from our center and those reported in the literature, we conclude that the phenotypic spectrum of *GRIN2B* is broad, which is characterized by DD/ID, epilepsy, hypotonia, language impairment, movement disorders, and behavioral disturbances. GOF variants were more likely to be located in the TMD region and to cause seizures and microcephaly than LOF variants. Additionally, memantine was effective in controlling seizures and improving cognition in patients with GOF variants. LOF missense variants caused more severe clinical phenotypes, including severe DD/ID, language delay, and movement disorders, than LOF truncated variants. This observation is consistent with the findings of other studies [42–45]. We hypothesize that the severe phenotype resulting from LOF missense variants in ion channel genes may be attributable to dominant-negative effects [42–45], however, more studies are needed to confirm it.

Grouping of patients based on the functional outcome (LOF or GOF) revealed clear and distinct phenotypic differences. First and foremost, GOF variants were predominantly located in the TMD regions, which may result in increased calcium influx and neuronal hyperexcitability [3, 46]. Secondly, patients carrying GOF variants were more likely to present with epilepsy and microcephaly than those with LOF variants. In line with our study, a previous study showed that TMD variants in *GRIN2A* primarily lead to GOF biophysical alterations [47]. Given that variants in the ABD region impair protein stability [36], our results showed that a higher proportion of LOF variants were located in the ABD region than GOF variants. However, there was no statistically significant difference. Thus, our findings provide clinical indicators that enable clinicians to predict probable GOF variants at an early stage and thereby provide appropriate treatment to patients.

We also analyzed recurrent variants to determine if they exhibited homogeneous phenotypes. However, the majority of the variants lacked consistent information, making it difficult to provide a reliable summary. Two males with the p.Met818Leu variant who presented with early-onset epileptic spasms, severe DD, hypotonia, and visual impairment achieved relief after memantine was administered. We observed that polymicrogyria and widened ventricles were the most common brain anomalies. These anomalies may be due to impaired neuronal migration caused by *GRIN2B* variants [48]. In addition to DD/ID and epilepsy, ASD was the most commonly observed phenotype, which may be due to structural abnormalities in neuronal synapses caused by *GRIN2B* variants [40].

An effective treatment option for *GRIN2B*-related epilepsy is still lacking. VPA, LTG, and TPM were the most commonly used ASMs in the seizure-controlled group, with treatment efficacies of 8.33%, 40.00%, and 30.00%, respectively. Three patients with infantile spasms used ACTH, and two of them experienced partial seizure control, which emphasizes the importance of ACTH therapy for this type of seizure, regardless of etiology [49, 50]. The antagonistic effect of memantine on NMDARs has been confirmed at the cellular level and in animal models [39, 51–53], nevertheless, the clinical therapeutic effect varies. Patients at our center with the p.Ile641Thr and p.Met818Leu variants experienced improvement after taking memantine. Another previous study found that memantine reduced convulsive seizures and electrographic epileptic discharges in patients with the *GRIN2A* p.Leu812Met variant [54]. However, it was ineffective in some cases, exacerbated seizures in patients with the p.Asn615Lys variant [54]. This suggests the presence of different mechanisms that are still unknown. To our knowledge, all seven patients (including those from the literature) with *GRIN2B* variants who were treated with memantine experienced seizure relief or cognitive improvement without any adverse effects. Otherwise, NMDAR agonists such as tobramycin and L-serine (approved by the FDA) [55] can be used to treat patients with LOF variants. Two studies have shown that L-serine improves cognition and movement in patients with LOF variants in the *GRIN2B* gene [15]. However, it is worth noting that seizures of our patient carrying the p.Met818Leu variant worsened after taking an NMDAR agonist (L-serine) and a AMPAR antagonist (perampanel). Therefore, clinicians should evaluate the functional changes of NMDAR receptors when prescribing L-serine or memantine. Although perampanel and memantine both target glutamate receptors, their efficacy on *GRIN2B*-related neurodevelopmental disorders varies. Therefore, perampanel might not be the best choice. Overall, our results highlight the benefits of memantine in treating *GRIN2B*-related epilepsy. Memantine demonstrated good tolerability in long-term treatment beginning from infancy, prompting its application to other *GRIN2B* GOF patients and subsequent translational studies investigating its mechanisms.

This study adds seven new cases harboring five novel *GRIN2B* variants. Moreover, it summarizes the phenotypic and genotypic features of an additional 98 cases of *GRIN2B*-related neurodevelopmental disorders from the literature. It highlights novel genotype-phenotype associations, which could be instrumental in enabling timely diagnosis and providing appropriate counseling for patients and their parents or guardians. The clinical phenotypes caused by *GRIN2B* LOF and GOF variants exhibit an overlap, including DD/ID, epilepsy, hypotonia, language impairment, movement disorders, and behavioral disturbances. Nevertheless, variants located in the TMD are more prone to being GOF and are more likely to cause epilepsy and microcephaly compared to LOF variants. Among LOF variants, missense variants lead to more severe clinical phenotypes than truncated variants, manifesting with severe DD/ID, absence of speech, and movement disorders. Our study emphasizes that memantine may effectively control seizures and improve cognition in patients with GOF variants.

However, our study has several limitations. Firstly, there exists a notable heterogeneity in the reported clinical data, particularly in the published cases. Secondly, electroclinical, neuropsychological, psychiatric, and drug response assessments were not systematically carried out across all patients. Furthermore, the methods used in this study for the evaluation of LOF and GOF variants are not universally applicable to all *GRIN2B* variants, such as insertions and copy number variations. Additionally, it remains unclear what factors contributed to the significant reduction in seizures observed in our patients treated with memantine. It is uncertain whether this improvement can be solely ascribed to memantine, the natural progression associated with the patients’ genetic variants (as seizures typically tend to ameliorate with age), or the combined effects of other therapeutic agents employed. We hope that our description of the genotypes and phenotypes will encourage the reporting of additional cases and facilitate the conduction of controlled clinical trials for this rare disease, which are of paramount importance for formulating more effective treatment strategies.

## Supplementary Information

Below is the link to the electronic supplementary material.

Supplementary Material 1

## Data Availability

Data of this study are available from the corresponding author upon reasonable request.
